# 711 Analysis of the Risk Factors for Mortality in Elderly Burn Patients

**DOI:** 10.1093/jbcr/irad045.187

**Published:** 2023-05-15

**Authors:** Wen-Teng Yao, Kwang-Yi Tung, Ying-Jun Liu

**Affiliations:** Mackay memorial hospital, Taipei, Taipei; Mackay memorial hospital, Taipei, Taipei; Mackay memorial hospital, Taipei, Taipei; Mackay memorial hospital, Taipei, Taipei; Mackay memorial hospital, Taipei, Taipei; Mackay memorial hospital, Taipei, Taipei; Mackay memorial hospital, Taipei, Taipei; Mackay memorial hospital, Taipei, Taipei; Mackay memorial hospital, Taipei, Taipei

## Abstract

**Introduction:**

The elderly experience higher mortality rates compared to younger burn patients with similar burn injuries. The aim of this retrospective study was to analyze the clinical factors affecting mortality in elderly burn patients admitted to single burn center.

**Methods:**

Data were collected on all elderly burn patients admitted to Mackay memorial hospital burn center between 2010 to 2020, focused on the population over 60 years old. The demographic data, burn profiles, hospital length of stay, use of mechanical ventilation and inotropic agents, delayed burn-related complication and hospital mortality were studied retrospectively. Both univariate and multivariate analysis were used to identify the risk factors for mortality.

**Results:**

61 elderly burn patients were evaluated and flame was the most common cause of burns(57.4%). All of the burns involved more than 20% of total body surface area. Sixteen patients(26.2%) expired during hospitalization. Univariate analysis showed that the mortality among elderly burn patients was associated with age(P< 0.001), burn surface area(P< 0.001), burn grade(P=0.016), use of inotropic agents(P< 0.001), initial endotracheal tube insertion(P< 0.001) and delayed burn-related acute kidney injury(P< 0.001), sepsis(P< 0.001) as well as pulmonary edema(P< 0.001). We stratified the above risk factors into two subgroups: Initial presenting patient profile(Age, TBSA, Burn grade) and delayed burn-related complication(Acute kidney injury, Sepsis, Pulmonary edema). Multivariate logistic regression was used to analyze the two subgroups. We found that in the initial presenting patient profile subgroup, age(P=0.014) and TBSA(P=0.004) were positively correlated to patient mortality. Conversely, among the burn-related complications subgroup, only sepsis(P=0.002) was the only significant independent factor related to patient mortality.

**Conclusions:**

This study identified key factors that affect mortality among elderly burn patients. Attention should be paid to those elderly burn patients with older age, increased TBSA, or delayed burn-related sepsis due to significantly increased mortality risk.

**Applicability of Research to Practice:**

Classic determinants of burn mortality are age, burn size, and the presence of inhalation injury (Revised Baux score). Several studies revealed that in-hospital complications was one of the strongest mortality predictors after burns. Understanding that sepsis is a major factor for mortality in elderly burn patient, we may improve our quality of care to decrease the mortality rate.

